# The Influence of Popular Beverages on Mechanical Properties of Composite Resins

**DOI:** 10.3390/ma14113097

**Published:** 2021-06-05

**Authors:** Leszek Szalewski, Dorota Wójcik, Marcin Bogucki, Jacek Szkutnik, Ingrid Różyło-Kalinowska

**Affiliations:** 1 Department of Integrated Paediatric Dentistry, Medical University of Lublin, 20-093 Lublin, Poland; 2 Department of Dental Prosthetics, Medical University of Lublin, 20-093 Lublin, Poland; dorota.wojcik@umlub.pl; 3 Department of Automation, Faculty of Mechanical Engineering, Technical University of Lublin, 20-618 Lublin, Poland; m.bogucki@pollub.pl; 4 Department of Functional Masticatory Disorders, Medical University of Lublin, 20-093 Lublin, Poland; jacek.szkutnik@umlub.pl; 5 Department of Dental and Maxillofacial Radiodiagnostics, Medical University of Lublin, 20-093 Lublin, Poland; rozylo.kalinowska@umlub.pl

**Keywords:** restorative dentistry, eating behaviours, aesthetic dentistry, biomechanics

## Abstract

Currently, composite resins are used in many restorative procedures. Previous studies showed that drinking beverages may affect the mechanical properties such as microhardness or flexural strength of dental composite resins. The aim of the present study was to investigate the influence of common beverages on the mechanical properties of composite resins. Samples of the materials were prepared according to the ISO 4049:2010 standard and producer’s recommendations. The samples were next conditioned in tested fluids: distilled water, sparkling water, Coca-Cola, Red Bull and orange juice for 7 days. Vickers microhardness and flexural strength testing was performed after 7 days. Performed statistic tests confirmed the significance of microhardness changes of the tested materials in terms of both different conditioning of the samples and different composite materials. The mean flexural strength of composites was highest in distilled water and it was reduced after one week in different beverages. We conclude that all tested beverages influenced on Vickers microhardness of tested composite resins. Flexural strength only in one material was statistically significantly influenced by tested beverages. The results of this study should be taken into consideration by a dentist preparing recommendations for the patients after dental treatment with usage of composite material or after cementing composite based fixed dentures.

## 1. Introduction

Composite materials are the most frequently used dental filling materials nowadays. They were introduced in the 1960s. Since then, their composition was modified to obtain the best mechanical and aesthetic properties. Nowadays, composite materials are used not only in conservative dentistry but also in prosthetics, periodontology and dental surgery. The veneers, crowns and composite abutments strengthened with glass or polyaramide fibres can meet the requirements of ceramic or metal-ceramic fillings [1,2].

Composite materials used in dental fillings are constantly exposed to noxious factors in the oral cavity which can change their basic properties. These factors can be classified as mechanical, thermal and chemical. Chemical factors can be divided into external (e.g., acids from the air, acidic nutritional products, chlorinated water in the swimming pool) and internal (gastric acids in frequent vomiting). These acids can cause erosive defects in tooth hard tissues and in composite materials.

Beverages have a different erosive potential dependent on the pH and titration acidity. Shaw and Smith defined the erosive potential of drinks [3]. They obtained the highest values for juices: orange, grapefruit and apple juice, and medium values for Coca-Cola, sparkling orange juice, and white wine. Sparkling water has a low erosive potential.

An average man should drink from 2 to 3 L of water per day [4]. The studies have shown that in Great Britain, half of this amount constitutes sweetened beverages, sparkling or isotonic drinks [5]. International reports show increasing consumption of such drinks among children [6]. According to the American Academy of Pediatrics Dentistry (AAPD) report, consumption of sweetened beverages in the United States has increased by 300% in the last 20 years. Increased consumption of sweetened beverages can cause a lot of adverse systemic effects, e.g., obesity. In the oral cavity, two types of negative effects can be observed: a low pH promotes the development of erosive defects in tooth hard tissue, whereas microorganisms metabolizing sugar cause enamel demineralization and caries. Hanging et al. proved that erosion depends on a liquid’s pH being between two and three, and only a slight decrease in pH value can increase the erosive potential of the liquids [7]. Due to these studies, a growing interest in developing methods of reduction of a liquid’s erosive potential is observed. Methods such as decreasing acid content, the addition of calcium, phosphorus or citrate compounds and green tea extract were investigated [5,8,9]. Unfortunately, these changes not only decreased acidity but also changed the taste of the beverages. 

Since composites have much lower tensile strength than compressive strength, and tensile strength is typically much more affected by internal flaws, this property is likely the most appropriate test of strength. However, flexure, a potentially simpler test method that relates well to tensile failure, is usually substituted in its place. Flexure testing is the standard means for strength testing of dental composites (ISO 4049) [10].

The basic aim of this study was to compare the stability of typical composite materials used in dentistry subjected to the negative impact of environmental factors. Their chemical composition reminds one of the common products included in patients’ diets. The stability of the materials was evaluated by Vickers microhardness and flexural strength. The null hypothesis was that different beverages do not affect the mechanical properties of dental composite resins (Vickers microhardness and flexural strength). 

## 2. Materials and Methods

### 2.1. Tested Materials

Characteristics of tested materials (all in A2 shade), including the composition of the filler, are shown in Table 1. The samples of the materials were prepared according to the ISO 4049:2010 standard [10] and producer recommendation. A total of 400 disc-shaped samples, 5 × 2 mm (Vickers microhardness test) and 400 rectangular specimens—25 × 2 × 2 mm (flexural strength test)—were prepared using a steel matrix. The matrix was placed on a glass slide to obtain a smooth surface. One portion of the composite material was imposed and condensed by conventional dental pluggers. The material surface was smoothed by pressing it down with a slide glass. Composite material polymerization was performed through the polyethylene film layer (40 µm) to eliminate oxygen inhibition on the surface. Polymerization (continuous mode, *t* = 20 s) was conducted as indicated by the producers and was performed using the same lamp for all the samples (Mini LED Standard, Acteon, France) with 1250 mW/cm^2^ output power. Prepared specimens were evaluated to eliminate samples containing air or other inaccuracies related to condensation and polymerization.

After polymerization, the samples were released from the matrix and stored in distilled water at 37 degrees Celsius for 24 h to simulate the conditions in the oral cavity. The samples were next randomly divided into subgroups, ten samples of each shape in each group, and conditioned in tested liquids: distilled water, sparkling water (Nałęczowianka, Nałęczów, Poland), Coca-Cola (Coca-Cola Company, Atlanta, GA, USA), Red Bull (Red Bull GmbH, Salzburg, Austria), and orange juice (Hortex, Warszawa, Poland) for 7 days. Table 2 presents the pH of tested beverages as calculated using the pH211 Microprocessor pH meter, which was manufactured by Hanna Instruments and calibrated using the Hamilton DuraCal buffer solutions (Hamilton Bonaduz AG, Bonaduz, Switzerland). Conditioning time was determined by guidelines from the study of Gawriołek et al. [11]. According to the schema, it was assumed that drinking one cup of coffee (150 mL) causes contact between the liquid and oral cavity lasting for 1 min. Assuming a daily consumption of 5 cups of beverage—750 mL—, a 7-day period of sample storage can be compared to a 5 year conditioning of the material in the oral cavity environment. All the samples were stored in a dark place at 37 degrees Celsius, in conditions resembling the oral cavity. The samples were next rinsed 5 times with distilled water and dried. The Vickers microhardness test and flexural strength test were performed on the polymerized side.

### 2.2. Vickers Microhardness Test

Microhardness of the tested samples was evaluated in Vickers Scale, using the durometer ZwickRoell ZHV 2 kg (ZwickRoell GmbH & Co. KG, Ulm, Germany) with a load force of 20 g (0.1962 N) working for 15 s. In case of an uneven or illegible probe footprint, the measurement was repeated. The diagonal length was measured using the light microscope Neophot 2 (Carl Zeiss AG, Oberkochen, Germany).

### 2.3. Flexural Strength Test

Flexural strength was measured using the ZwickRoell Z010 (ZwickRoell GmbH & Co. KG, Ulm, Germany) testing machine with the opening width of 20 mm, initial gripping force of 1 N and crosshead speed of 0.75 mm/min. The specimens were measured to an accuracy of 0.01 mm before the test. Its end was marked by the specimen being crushed. Flexural strength was calculated using the following Equation (1):

S = 3FL/(2BH^2^) (1)

where F is the maximum load in Newtons exerted on the specimens, L is the distance (20 ± 0.01 mm) between the supports, B is the width (2 ± 0.01 mm) of the specimens measured immediately prior to testing, and H is the height (2 ± 0.01 mm) of the specimens measured immediately prior to testing.

### 2.4. Statistical Methods

For the microhardness test, experimental systems of the study were a combination of 2 investigated factors: type of material (eight-level variability) and used liquid (five-level variability). The scheme and experiment implementation was a two-factor, complete, randomized plan. The two-way analysis of variation was used to perform a statistical elaboration of the study.

A fixed-effects model was postulated. The effects were related to microhardness measurements caused by a different:

1. Composite material;
2. Conditioning environment;
3. Interaction of both factors.

In addition, Tukey’s post hoc tests were used to compare measurements within each experimental system. The hypotheses referring to the postulated empiric model and compared median values were verified and the probability value *p* = 0.05 was assumed to be the level of significance of the statistical tests. It refers also to additional statistical tests performed to verify the propriety of an empiric model structure.

Means and standard deviations of flexural strength were calculated. The Shapiro–Wilk test was used to measure the normality of distribution in individual groups, whereas the homogeneity of variances was assessed using the Levene’s test. The following tests were applied to determine the significance of differences between the groups: a parametric test of a one-way analysis of variance, a non-parametric Kruskal–Wallis ANOVA test, and a Tukey’s post hoc range test (honest significant difference). The level of significance was set at α = 0.05.

## 3. Results

The results of the measurements of material microhardness are shown in Table 3. The results allowed to create the variance model (section “Statistical methods”). It should be mentioned that before basic calculations and statistical analyses, the microhardness measurements were initially selected and extreme observations were excluded.

Performed statistical tests confirmed the significance of microhardness changes of the tested materials, both in terms of conditioning and material composition. The interaction between investigated factors was also proven. All the observed effects are shown in Table 3. According to the results, changes in the environment caused a statistically significant change in microhardness of the sample in most cases. This conclusion refers especially to such materials as GrandioSO, Polofil-Supra, G-aenial and Gradia Direct Anterior. Microhardness changes show statistically significant differences for these materials. GrandioSO is a composite material that was the most resistant to the environmental factors (about 55 HV on average). Materials such as Boston, F2, Polofil-Supra and Arabesk had a similar, but not so high, resistance (microhardness about 40 HV). It should be noted that three materials—F2, Polofil-Supra and Arabesk—showed a comparable, slight dispersion of average values. It proves good composition of the materials, designed to obtain an equal mechanical resistance (microhardness) despite the environment. The rest of the materials showed average or low resistance to microhardness changes (Kalore—32 HV; G-eanial—24 HV; and Gradia Direct Anterior—20 HV). The results presented in Table 3, confirmed by a proper statistical analysis, show that orange juice was the most unfavourable conditioning environment, followed by Coca-Cola, Red Bull, sparkling water and distilled water, respectively. 

Table 4 presents the mean values of the specimens’ flexural strength after the 7-day immersion in the tested beverages. The mean flexural strength values of the different tested composites after immersing in different beverages were slightly lower than in the control group (distilled water) except for the mean flexural strength of the Boston material immersed in sparkling water. However, the flexural strength of Gradia Direct Anterior displayed a statistically significant difference (*p* < 0.05) concerning the specimens stored in Coca-Cola and orange juice and, in these cases, durability was lower than in the control group. Other tested composite resins did not show any statistically significant differences with regards to flexural strength after incubation in the beverages.

## 4. Discussion

The tested beverages decreased the microhardness of dental composite resins. Flexural strength of Gradia Direct Anterior was statistically significantly influenced by tested beverages. Hence, the null hypothesis was rejected for microhardness of dental composite resins.

Product characteristics describe mechanical properties of materials according to the standards, e.g., PN-EN ISO 4049. Test results defined by the standards do not correspond with the clinical situation due to differences between the oral cavity environment and the environment in vitro. Unfortunately, these data do not include information about the impact of erosive factors from a diet on composite material properties. The modern diet contains beverages and foods with a low, or even very low, pH, e.g., Coca-Cola—2.42 or orange juice—3.86. Frequent consumption of these products can cause deterioration of mechanical properties of the composite material and lead to the destruction of prosthetic work and fillings of the cavities. Weakened composite materials can easily break, cause the unsealing of fillings and lead to secondary caries development.

Appropriate material properties can be obtained by using different types of the filler. Composite materials with microfillers provide a good aesthetic effect and can be easily polished, although they have worse mechanical properties in comparison to other composite materials. Because of that, they are mostly used in front teeth fillings where high bite forces are not observed. In lateral regions with bite forces of 500–600 N [12] or even 847 N [13], better mechanical durability provided by hybrid (microfiller–macrofiller connections) and nanohybrid fillings are required.

Our own research revealed that the Gradia Direct Anterior, which is a micro-hybrid material with a large part of micro filler, has the lowest microhardness. The highest microhardness was found for the GrandioSO composite materials with nanofiller. In our study, we did not obtain statistically significant differences between the micro and nano-hybrid groups. The tested materials differed not only in the size of the filler, but also in its chemical composition. Additionally, there were differences in resins which could also influence the obtained results. In order to assess how the size of the filler affects the mechanical properties, it would be necessary to create a test material containing the same resins and differing only in the size of the filler having the same chemical composition.

Yanikoglu et al. [14] demonstrated that samples conditioned in distilled water show lower microhardness values in Vickers scale than samples kept dry. Our research was carried out in vitro, so its relation to clinical conditions is not fully objective. The impact of the beverages is temporary, it can only be prolonged in tooth pockets or adjacent to leaking fillings. It should be noted that teeth and fillings are protected by saliva, buffer substance favourably changing the environmental pH. Chemical factors play a fundamental role in the degradation process of the composite resin’s surface. The pH is a very important factor to determine the erosive potential of a solution. Hwang et al. found that the lower the pH of the solution, the greater the decrease in microhardness of the composite material [15]. The large decrease in microhardness may be related to the increase in surface roughness under the influence of beverages. This can be prevented by properly polishing the dental composites.

The studies showed the significant impact of beverage composition on the erosion of the composite materials, while the protective function of saliva was omitted. The producers do not include any information on the negative impact of the beverages on their products. The results show a significant correlation between the consumption of beverages and microhardness reduction, promoting the destruction of filling and prosthetic work. This phenomenon can be observed less frequently in clinical conditions. However, not only erosive factors correlated with diet but also other factors such as bite forces, endogenic acids and mechanical damage resulting from bad hygienic habits can be observed. 

Our research confirmed the results of Yanikoglu et al. [14] concerning the impact of substances contained in Coca-Cola on Vickers microhardness reduction. Moreover, the cited authors proved the correlation between this effect and the conditioning time—the samples were investigated after 24 h, 30 days and a year. Similar results were obtained by Tanthanuch [16] analysing the impact of apple cider, orange juice, Coca-Cola, coffee and beer on nanohybrid resin composites. They showed that microhardness values of all groups decreased from the initial week of immersion until the end of the 28-day period, and the greatest shown change in hardness occurred within the first 7 days. Information on microhardness reduction of the material depending on conditioning time has a great clinical implication, because it shows the negative impact of excessive consumption of sweetened beverages with a low pH such as fruit juices or sweetened sparkling beverages. Aliping-McKenzie et al. [17] reports that microhardness of composite materials decreased after sample storage in Coca-Cola, orange and apple juice. It should be noted that the components of fruit juices cause more pronounced changes of microhardness than sparkling beverages. On the other hand, the decrease in microhardness of composite materials under the influence of alcoholic drinks was related to the alcohol content and not to the pH of the liquid [18]. Similar results were obtained by Colombo et al. investigating new composite materials enriched with ceramic used in CAD/CAM systems. Despite the initial high values of microhardness under the influence of Coca-Cola, they also decreased [19]. The studies on the influence of coffee components on the properties of composite materials showed changes in sample colour, but not in their microhardness [20].

In spite of the query of databases such as PubMed, Scopus and Science Direct, the authors did not find many studies on the influence of energy drink components on the properties of composite materials. It is an important dental problem in terms of the increasing consumption of energy drinks among youth. Energy drinks are consumed by 30%–50% of adolescents, with 31% of 12–19-year-olds admitting to regular ED consumption [21]. In the study of Nowak and Jasionowski, 67% of 12–20-year-old students (*n* = 2629) consumed energy drinks regularly [22]. Our research confirmed the impact of Red Bull components on significant reduction of microhardness of materials: Gradia Direct Anterior, G-aenial, Kalore and Boston. The studies of Erdemir et al. [23,24,25] investigating the impact of energy drink components on the properties of composite materials confirms these results. These authors proved that energy drinks can cause changes of colour and microhardness of the composite materials, irrespective of the filling used. 

There are numerous studies on composite aging, measuring how mechanical properties of composites in the human oral cavity change over time. Janda et al. [26] examined 12 different composites in a flexural strength test. Two of the materials tested did not even meet the required norms, so their results were below 80 MPa. After aging the specimens using thermocycles, there was one more material that did not meet the norms. As observed by Stawarczyk et al. [27], after 180 days of thermocycles, the flexural strength of Gradia Direct Anterior changed from 73 to 66 MPa; therefore, it was below the norm. 

As observed by Schmidt and Ilie [28], as well as Yanikoglu et al. [14], composite specimens change their mechanic properties when stored in distilled water. Schmidt and Ilie [28] tested six different composites with a nanofiller immersed in distilled water, artificial saliva and alcohol. Although the biggest changes were observed in the third case, differences were also noticeable in the case of specimens immersed in distilled water and artificial saliva. 

In our study, the mean values of flexural strength of seven from eight composites immersed in beverages for seven days are beyond the level of statistical significance, which is also confirmed in other studies [29,30]. However, it is worth noting that the decrease occurred and could be measured by calculating mean values of flexural strength. Based on other authors’ observations, it can be concluded that longer immersion in the beverages would result in statistically significant changes [28]. On the other hand, Scribante et al. obtained a statistically significant decrease in flexural strength of composite resin after soaking in Coca-Cola after 7 days [31,32]. 

Flexural strength was not statistically significantly different after a seven-day immersion in citric acid 0.1 M, lactic acid 0.1 M, heptane and ethanol [29,30]. Longer incubation of the specimens in alcohol—2 or 4 weeks—causes statistically significant decrease in flexural strength [28]. Similarly, Jyothi et al. [33], who tested mouthwashes, also confirmed alcohol’s negative influence on composites. This is particularly important in view of the increasing popularity of mouthwashes among patients. 

Our research was conducted in vitro conditions, so its results can hardly be extrapolated into clinical situations. Exposure to beverages is temporary, and the contact may be longer only in natural tooth hollows or in the area of leaky overhanging fillings. Otherwise, teeth and fillings are protected by saliva, which is a buffer that changes the environment’s pH into a favourable one. However, apart from the erosive contents in food, numerous other factors occur within the oral cavity, e.g., occlusal forces, endogenic acids or mechanic damages resulting from inappropriate health habits. Since many patients may not be aware of the impact of an unhealthy diet on the newly restored teeth, the above-mentioned factors should also be considered by dentists when instructing a patient with a composite filling or prosthesis. 

The components of the beverages can also cause changes of other properties of composite materials, such as the elasticity module [34], roughness of the surface [35] or solubility [36].

## 5. Conclusions

Within the limitations of this in vitro study, it may be concluded that all tested beverages influenced on Vickers microhardness of tested composite resins. Flexural strength only in one material was statistically significantly influenced by common drinks. The results of our own research have shown substantial differentiation of mechanical properties of the materials used in conservative dentistry nowadays, depending on different conditioning environments. The results can influence the choice of the material used by a dentist, after taking into account the patient’s eating habits. The most unfavourable conditioning environment was orange juice, which caused the greatest mechanical properties reduction of the examined samples, followed by Coca-Cola, Red-Bull, sparkling water and distilled water as a control group. The results of this study should be taken into consideration by dentists preparing recommendations for their patients after filling cavities with the composite material or cementing composite fixed dentures. It would be advisable to create a new material resistant to the influence of popular beverages.

## Figures and Tables

**Table 1:** Compositions of the resin materials tested in a table.

Name	Company	Filler Type	Filler Size	% of Total Weight
Gradia Direct Anterior	GC E, Belgium	Micro-hybrid	Silica—0.85 µm	73%
G-aenial	GC EUROPE, Belgium	Nano-hybrid	Silica/strontium glass, lanthanum fluoride 16–17 µm, silica > 100 nm, colloidal silica < 100 nm	89%
Kalore	GC EUROPE, Belgium	Nano-hybrid	Prepolymerized filler 17 µm strontium glass, lanthanum fluoride > 100 nm, colloidal silica < 100 nm	82%
Boston	Arkona, Poland	Micro-hybrid	Barium–aluminium–silicon glass, fumed silica, titanium dioxide 0.72 µm	78%
F2	Arkona, Poland	Micro-hybrid	Fluorine–barium–aluminium–silica glass exerting fluorine, fumed silica, titanium dioxide 0.90 µm	79%
GrandioSO	VOCO GmbH, Germany	Nano-hybrid	Nanoparticles 20–40 nm, glass–porcelain material 0.05 μm	89%
Polofil Supra	VOCO GmbH, Germany	Micro-hybrid	Sintraglass system (microfiller 0.05 μm macrofiller 0.5–2 μm)	76.50%
Arabesk	VOCO GmbH, Germany	Micro-hybrid	Sintraglass system (microfiller 0.05 μm macrofiller 0.5–2 μm)	76.50%

**Table 2:** Beverages tested and their pH values.

Distilled Water (Control Group)		Sparkling Water		Red Bull		Coca Cola		Orange Juice	
pH	Temp (°C)	pH	Temp (°C)	pH	Temp (°C)	pH	Temp (°C)	pH	Temp (°C)
5.83	21.7	5.41	22.2	3.41	22.3	2.42	21.3	3.86	21.4
5.82	21.9	5.45	22.2	3.42	22.5	2.42	21.3	3.87	21.5
5.83	22	5.45	22.4	3.41	22.3	2.42	21.7	3.86	21.5

**Table 3:** A two-factor factorial design along with the statistics of particularly experimental sets (means of Vickers Microhardness (HV) measurements and their corresponding standard errors (SE)).

Composite Material	Distilled Water (Control Group)		Sparkling Water		Coca-Cola		Red Bull		Orange Juice	
	Mean	SE	Mean	SE	Mean	SE	Mean	SE	Mean	SE
Gradia Direct Anterior	26.60	0.55	21.54 *	0.26	17.09 *	0.25	16.17 *	0.22	16.37 *	0.27
G-aenial	30.25	0.39	24.22 *	0.20	22.03 *	0.25	22.64 *	0.23	20.88 *	0.20
Kalore	37.80	0.60	33.27 *	0.52	28.18 *	0.28	32.48 *	0.34	23.95 *	0.26
Boston	45.11	0.52	39.85 *	0.41	29.77 *	0.34	36.34 *	0.45	36.33 *	0.37
F2	46.22	0.37	45.35	0.45	39.26 *	0.40	39.18 *	0.32	38.28 *	0.40
Polofil Supra	45.99	0.49	39.69 *	0.37	37.68 *	0.33	39.07 *	0.34	36.70 *	0.34
GrandioSO	61.73	0.69	54.53 *	0.58	51.92 *	0.62	56.58 *	0.54	47.33 *	0.43
Arabesk	39.99	0.39	36.79 *	0.36	36.07 *	0.34	37.30 *	0.33	35.45 *	0.32

* Statistically significant difference (*p* < 0.05) to the control group.

**Table 4:** The mean flexural strength (FS) values (MPa) with standard deviations.

Composite Material	Distilled Water (Control Group)		Sparkling Water		Coca-Cola		Red Bull		Orange Juice	
	Mean	SD	Mean	SD	Mean	SD	Mean	SD	Mean	SD
Gradia Direct Anterior	89.35	7.91	87.69	12.2	76.51 *	7.21	83.48	5.36	77.64 *	8.39
G-aenial	98.44	6.42	94.70	6.57	89.24	10.96	98.40	10.71	94.55	10.93
Kalore	95.71	19.7	94.26	14.8	88.47	8.76	90.00	12.63	93.27	11.91
Boston	127.95	16.51	128.30	11.98	117.32	22.29	127.58	17.98	127.61	23.67
F2	131.23	28.04	130.20	20.45	122.71	26.04	117.05	19.08	122.60	13.38
Polofil Supra	139.90	19.38	128.47	22.14	113.52	27.67	134.10	22.72	124.65	29.84
GrandioSO	136.58	34.65	133.66	29.15	126.60	27.82	131.17	27.87	133.31	31.34
Arabesk	132.06	19.59	117.74	22.49	105.96	28.18	117.75	27.78	110.95	25.64

* Statistically significant difference (*p* < 0.05) to the control group.

## Data Availability

The data that support the findings of this study are available from the corresponding author L.S., upon reasonable request.
